# Systematic Review, Meta-Analysis and Bioinformatic Analysis of Biomarkers for Prognosis of Malignant Pleural Mesothelioma

**DOI:** 10.3390/diagnostics12092210

**Published:** 2022-09-12

**Authors:** Zhenhua Lu, Wenlong Zhang, Ke Huang, Mucheng Zhu, Xiaoting Gu, Defang Wei, Mingxuan Shi, Yaqiong Chen, Huihui Wang

**Affiliations:** 1 School of Stomatology, Lanzhou University, No. 222 Tianshui South Road, Lanzhou 730030, China; 2 School of Basic Medical Sciences, Lanzhou University, No. 222 Tianshui South Road, Lanzhou 730030, China; 3 First School of Clinical Medicine, Lanzhou University, No. 222 Tianshui South Road, Lanzhou 730030, China; 4 Second School of Clinical Medicine, Lanzhou University, No. 222 Tianshui South Road, Lanzhou 730030, China

**Keywords:** MPM, biomarkers, meta-analysis, prognosis, bioinformatics analysis

## Abstract

In previous studies, non-invasive diagnostic biomarkers showed great benefit in the early-stage diagnosis of malignant pleural mesothelioma (MPM). However, the accuracy of different biomarkers was controversial. In this study, meta-analysis and bioinformatics analysis were conducted to compare the accuracy of the following three biomarkers and explore the relationship between the gene expression levels and MPM. A systematic search of meta-analysis was conducted using PubMed, EMBASE and Cochrane Library to identify relevant studies from the inception to March 2021. QUADAS-2 for Quality Assessment of Diagnostic Accuracy Studies was used to evaluate the quality of eligible studies. The meta-analysis was performed utilizing Stata 15.0 and Review Manager 5.4 software. The meta-analysis results showed that 31 studies that involved 8750 participants were included. The pooled sensitivity and specificity (SPE) were 0.90 (95% CI: 0.74, 0.97) and 0.91 (95% CI: 0.84, 0.95) for Fibulin-3, 0.66 (95% CI, 0.51–0.78) and 0.91 (95% CI, 0.82–0.96) for mesothelin (MSLN), 0.68 (95% CI: 0.63,0.73) and 0.86 (95% CI: 0.82,0.90) for soluble mesothelin-related peptides (SMRP), and 0.74 (95% CI, 0.66-0.80) and 0.89 (95% CI, 0.85–0.91) for MSLN + SMRP + Fibulin-3. Compared with the other two biomarkers, Fibulin-3 may be more appropriate to be one of the indicators for combined diagnosis. Bioinformatics analysis showed that the low expression level of the MSLN gene was significantly related to longer survival time and better prognosis of MPM patients. However, considering the limitation in the quality and sample size of the included research, further studies are required.

## 1. Introduction

Malignant pleural mesothelioma (MPM), an aggressive and highly fatal tumor primarily caused by exposure to asbestos, mostly come from a series of cells on the surface of the pleura, and a small part from the peritoneum and pericardium [1]. The incidence of MPM has been increasing in recent years. An estimated 1000 people die annually from MPM between 2010 and 2020 [2,3,4,5]. Due to the long incubation period and no specificity of symptoms, the prognosis of patients is poor [6]. Currently, there is still much research space for the treatment of MPM and its median survival time is 9.2–14 months [7,8]. It is worth noting that if the tumor is removed as early as possible, the survival of patients with early diseases will be prolonged to some extent [9,10,11]. Although mesothelioma treatment does not significantly prolong life, early diagnosis of MPM can strive for a certain time for subsequent treatment [12]. Therefore, it is urgent to find accurate means to identify MPM in the early stage.

So far, the primary diagnostic method of MPM is the histopathological assessment of pleural biopsy [13,14]. In addition, the examination results must be explained from the perspective of morphology, which has certain limitations [15]. Therefore, tumor biomarkers have attracted more and more attention due to their less invasive features. Exploring the biomarkers of MPM is helpful for the screening and early diagnosis of MPM and improves the prognosis. At the same time, by looking for suitable methods, taking the correlation between easily detectable biomarkers and patient survival as an entry point, we can work together from the three aspects of disease diagnosis, treatment and prognosis to strive for better treatment effects.

To diagnose MPM the early phase, the more widely studied biomarkers are Mesothelin (MSLN), soluble mesothelin-related peptide (SMRP), Osteopontin (OPN), calretinin and Fibulin-3 [16,17,18]. In recent years, proteins of SMRP, MLSN and Fibulin-3 have received great attention for the diagnosis of MPM [19,20]. Previous studies showed that SMRP has been proved to have reasonable specificity and good diagnostic effect [21]. However, SMRP is not unique to MPM and has been widely studied as an early biomarker for contacting asbestos [22,23,24]. As a cell surface glycoprotein with the function of adhesion between cells [21], MSLN is highly expressed in many cancers, such as MPM, ovarian and pancreatic cancers [25,26]. Fibulin-3 is an extracellular glycoprotein widely expressed throughout the body and adult tissues during development.

In general, three common biomarkers for diagnosing of MPM have been widely studied, but their sensitivity and specificity are greatly limited due to the heterogeneity between different research types. In the present study, we conducted a meta-analysis based on all the research data of the three proteins for the diagnosis of MPM, aiming to find out the best biomarkers to diagnose MPM with higher accuracy, and strive for valuable time for subsequent symptomatic treatment and prognosis. In addition, relevant data from The Cancer Genome Atlas (TCGA) were collected to explore the relationship between the gene expression levels of the three antibodies edited MSLN, SMRP, Fibulin-3 and the prognosis of MPM at the molecular level. Based on the relationship between the expression of the corresponding gene and prognosis, according to the tumor TNM stage, different subgroups were formulated for rational analysis, and some new findings were obtained.

## 2. Materials and Methods

This study was conducted following the Preferred Reporting Items for Systematic Reviews and Meta-Analyses (PRISMA) guidelines, registered on INPLASY [27] (registration number: INPLASY202230124), and is available in full on inplasy.com (https://inplasy.com/inplasy-2022-3-0124/; accessed on 23 March 2022).

### 2.1. Search Strategy and Study Selection

**Figure 1:**
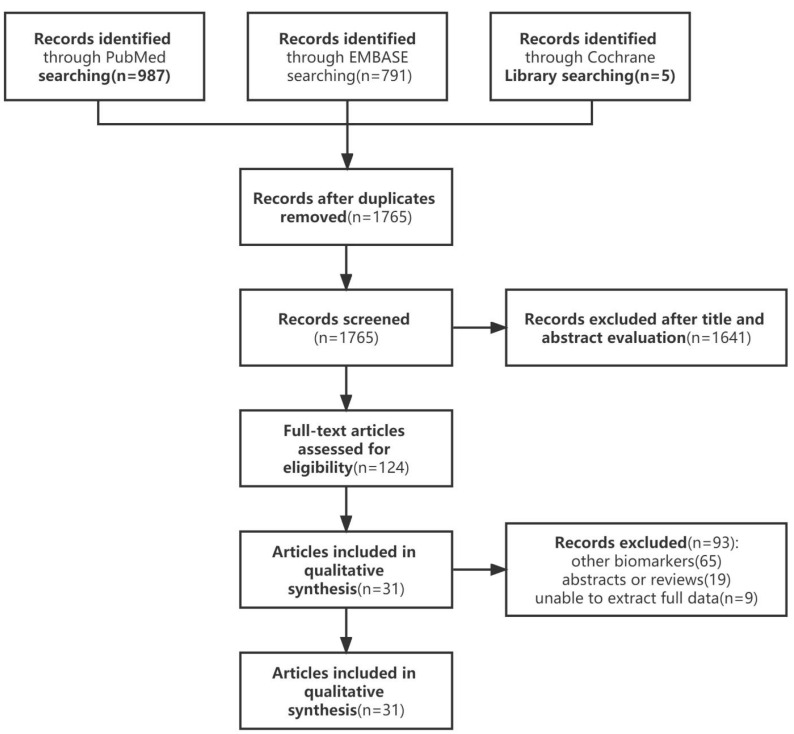
Flowchart of literature search.

Until March 2021, a systematic search was conducted in PubMed, Embase, and Cochrane Library. Figure 1 and Table 1 list the details of the literature retrieval strategy. 

Articles that meet the following inclusion criteria are considered eligible for selection: (a) Study type: We evaluated the diagnostic accuracy of MPM protein markers prospectively or retrospectively. There were no restrictions on quality, sample size or number of patients. (b) Participants: Patients diagnosed with MPM by histopathological examination were included, excluding those with distant metastasis of MPM. Some studies collected and analyzed samples before diagnosis, but after subsequent checking with the gold standard, we included only patients with a confirmed diagnosis of MPM. There are no restrictions on race, sex, age, or cancer stage. (c) Reference criteria: Pleural biopsy tissue obtained surgically for histopathological diagnosis. (d) Outcomes: The area under the curve (AUC), Sensitivity (SEN), specificity (SPE), diagnostic odds ratio (DOR), positive likelihood ratio (PLR), negative likelihood ratio (NLR). Exclusion criteria: (a) animal studies; (b) articles not published in English or Chinese; (c) conference abstracts, meta-analyses, reviews, case reports, letters, duplicates, expert opinion, or multiple publications; (d) not enough data can be extracted to calculate sensitivity and specificity.

When the search was completed, the title and abstract of each study were screened independently by two authors. We obtain all articles deemed appropriate by any party in the full text for further evaluation. Then, the same two authors will evaluate potential full texts and select studies based on inclusion/exclusion criteria, discuss the included studies and reach agreement to resolve differences through discussion and consensus. If no agreement can be reached, the opinion of the third reviewer will be sought.

### 2.2. Data Characteristics and Quality Assessment

A literature search was conducted by two independent reviewers to assess eligibility for each study. The third researcher solved conflict problems. This article reviews the titles, abstracts and full texts of all relevant studies, the following information is taken from all eligible articles: (a) Basic information: author, number of authors, publication year, journal name, country of the journal, country of the corresponding author, funding, and types of included studies; (b) Sample size: number of included studies; (c) Baseline characteristics: baseline diagnosis, sex, age, and location; (d) The index tests: number and name of biomarkers; (e) Data of SEN, SPE, NLR, DOR, AUC, and their 95% CI of each original study included in the article.

### 2.3. Risk of Bias and Quality of Evidence

Quality was assessed using the revised Diagnostic Accuracy Research Quality Assessment Tool (QUADAS-2) (HTA programme 2011 (www.hta.ac.uk)). The tool is evaluated in terms of patient selection, indicator testing, reference criteria and patient flow through the study, and timing of indicator testing and reference criteria. The answer to each question was “yes”, “no” or “unclear.” Concerns about applicability were rated as “low”, “high” or “unclear” [57].

### 2.4. Assessment of Publication Bias

Deek’s funnel plot was conducted to detect publication bias where there were more than 10 studies available for an index test.

### 2.5. RNA-seq Data Acquisition and Survival Analyses

The RNA-seq data consisted of 86 tumor tissues and corresponding clinical information was collected from TCGA. Clinical information included tumor stage, histological subtype (epithelioid, sarcomatous, biphasic), age, and sex. Samples with unclear information were excluded. Patients were split into two groups according to the median expression level of the target gene. Kaplan–Meier (KM) survival analyses were carried out using the R package (survminer, v.0.4.9 and survival, v.3.2.10) (https://CRAN.R-project.org/package=survminer) (http://cran.r-project.org/package=survival).

### 2.6. Univariate and Multivariate Cox Regression Analyses

Univariate and multivariate Cox regression analyses were carried out, using R package (survminer, v.0.4.9 and survival, v.3.2.10), to figure out the prognostic role of the target gene.

### 2.7. Statistical Analysis

In this article, Stata 15.0 (Stata Corporation, College Station, TX, USA) and Review Manager 5.4 statistical software programs were used to test the heterogeneity of the research and perform meta-analysis. We obtained a 2 × 2 contingency table by extracting the sensitivity and specificity data of each study. The SEN, SPE, PLR, NLR, and DOR of the study are calculated, and the SROC curve was generated. The statistical calculations of data from TCGA were processed through R software (v.3.6.3), Vienna, Austria.

## 3. Results

### 3.1. Search Results and Quality Assessment

The literature retrieval process was shown in Figure 1. The selected studies were published between 2003 and 2018, including 1950 cases of MPM patients and 6800 cases of non-MPM patients. After systematic retrieval, we obtained 1783 studies, removed eighteen duplicates, reviewed titles and abstracts, and excluded 124. After reading the full text, we excluded 93 studies that were not related to the research content of this paper, and finally obtained a total of 31 articles [17,24,28,29,30,31,32,33,34,35,36,37,38,39,40,41,42,43,44,45,46,47,48,49,50,51,52,53,54,55,56] that met the requirements. Table 1 summarizes the characteristics of the included studies. All MPM patients are diagnosed by cytology and histopathology.

The methodological quality of the study was assessed by QUADAS-2. The results showed that the quality of the studies was all satisfactory, which made the final analysis data more reliable. The quality of the included studies is summarized in Supplementary Figure S1A. Detailed information on the risk of bias and applicability issues for each included study is provided in Supplementary Figure S1B.

### 3.2. Diagnostic Accuracy

**Figure 2:**
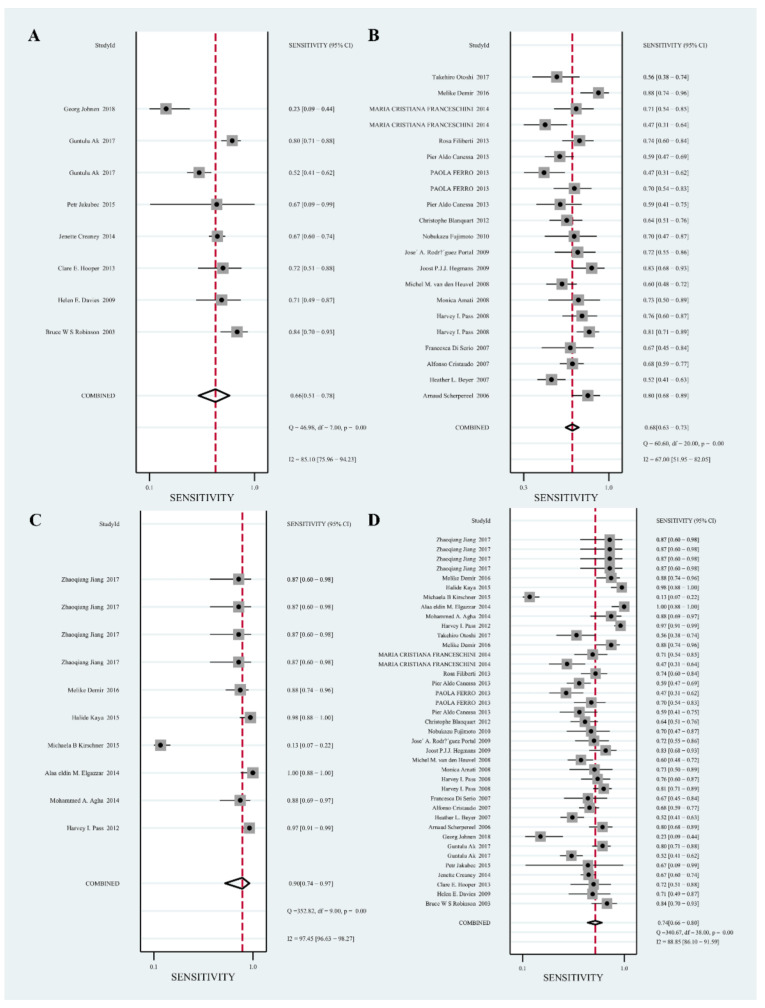
Forest plot for the pooled sensitivity (SEN) of the 3 biomarkers. (**A**): Mesothelin (MSLN) [29,30,31,32,33,34,35]; (**B**): soluble mesothelin-related peptides (SMRP) [24,37,42,43,44,45,46,47,48,49,50,51,52,54,55,56,57]; (**C**): Fibulin-3 [17,47,48,51,52,54]; (**D**): MSLN + SMRP + Fibulin-3 [17,24,28,29,30,31,32,33,34,35,36,37,38,39,40,41,43,44,45,46,47,48,49,51,52,53,54,55,56].

**Figure 3:**
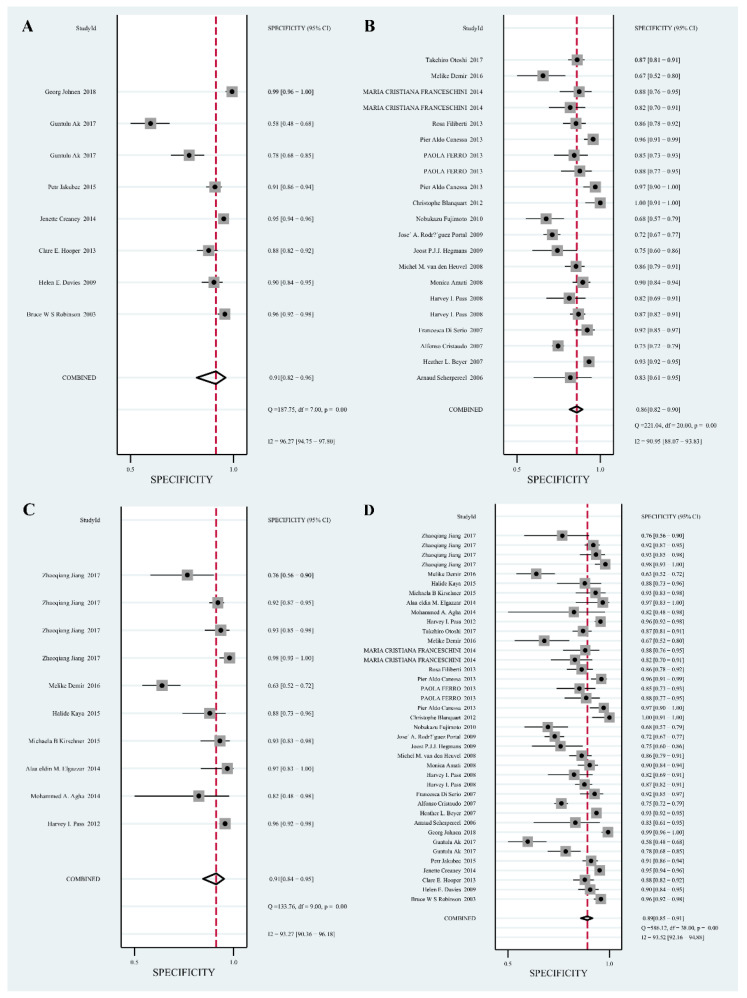
Forest plot for the pooled specificity (SPE) of the 3 biomarkers. (**A**): Mesothelin (MSLN) [29,30,31,32,33,34,35]; (**B**): soluble mesothelin-related peptides (SMRP) [24,29,30,31,32,33,34,35,36,38,39,40,41,43,45,52,55]; (**C**): Fibulin-3 [17,47,48,51,52,54]; (**D**): MSLN + SMRP + Fibulin-3 [17,24,28,29,30,31,32,33,34,35,36,37,38,39,40,41,43,44,45,46,47,48,49,51,52,53,54,55,56].

The pooled SEN and SPE results of the 3 biomarkers were shown in Figure 2 and Figure 3. The forest plot of meta-analysis shows that MSLN had a pooled SEN of 0.66 (95% CI, 0.51–0.78), a pooled SPE of 0.91 (95% CI, 0.82–0.96); pooled SEN of 0.68 (95% CI, 0.63–0.73) and pooled SPE of 0.86 (95% CI, 0.82–0.90) for SMRP; pooled SEN of 0.90 (95% CI, 0.63–0.73) and pooled SPE of 0.91 (95% CI, 0.82–0.90) for Fibulin-3. The forest plot of the meta-analysis shows that MSLN + SMRP + Fibulin-3 had a pooled SEN of 0.74 (95% CI, 0.66–0.80) and a pooled SPE of 0.89 (95% CI, 0.85–0.91).

**Figure 4:**
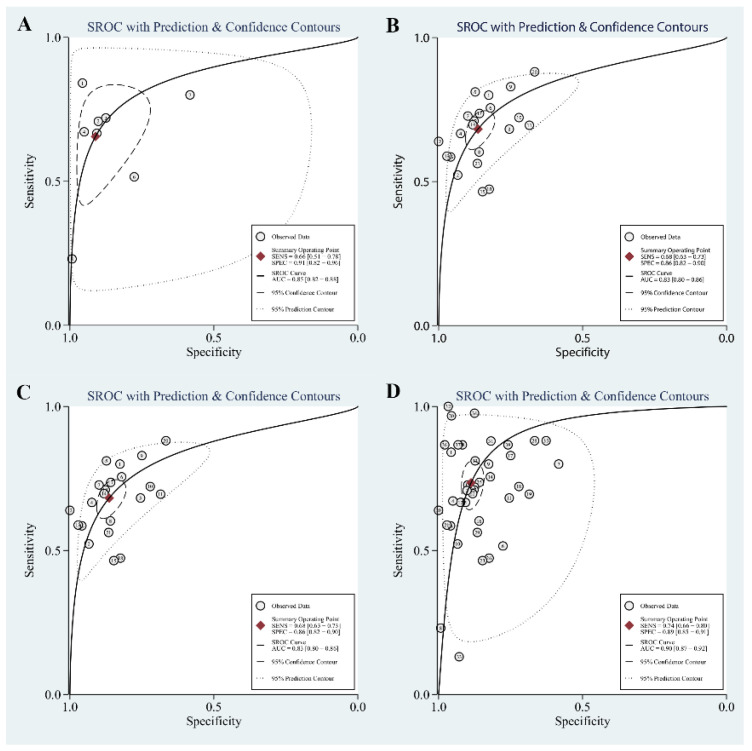
The area under the curve (AUC) of the 3 biomarkers. (**A**): Mesothelin (MSLN); (**B**): soluble mesothelin-related peptides (SMRP); (**C**): Fibulin-3; (**D**): MSLN + SMRP + Fibulin-3.

The area under the SROC curve is shown in Figure 4. The area under the SROC curve was 0.85 (95% CI: 0.82–0.88) for MSLN, 0.83 (95% CI: 0.80–0.86) for SMRP, 0.96 (95% CI, 0.93–0.97) for Fibulin-3, and 0.90 (95% CI, 0.87–0.92) for MSLN + SMRP + Fibulin-3. The data above show that Fibulin-3 had the highest diagnostic accuracy in the diagnosis of MPM compared to other biomarkers.

### 3.3. Prognostic Analysis of MSLN Gene in Mesothelioma

**Figure 5:**
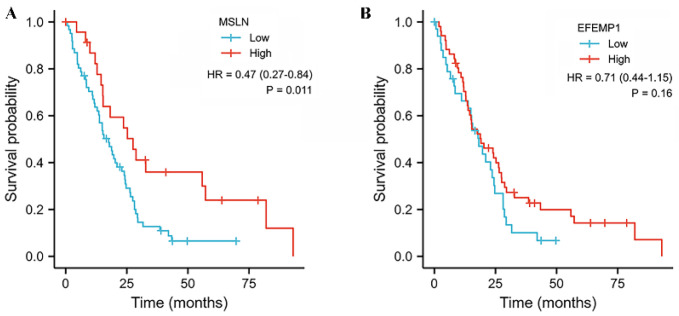
Overall prognostic analysis of MSLN (**A**) gene and EFEMP1 (**B**) gene in mesothelioma in mesothelioma.

The overall prognosis analysis of MSLN gene in mesothelioma, the Log-rank test results showed a significant difference in survival time between high and low MSLN gene expression groups (*p* = 0.011). The results showed that in MPM patients, the higher the MSLN gene expression, the longer the patient’s survival and the better the prognosis. The results are shown in Figure 5.

### 3.4. Subgroup Analysis

**Figure 6:**
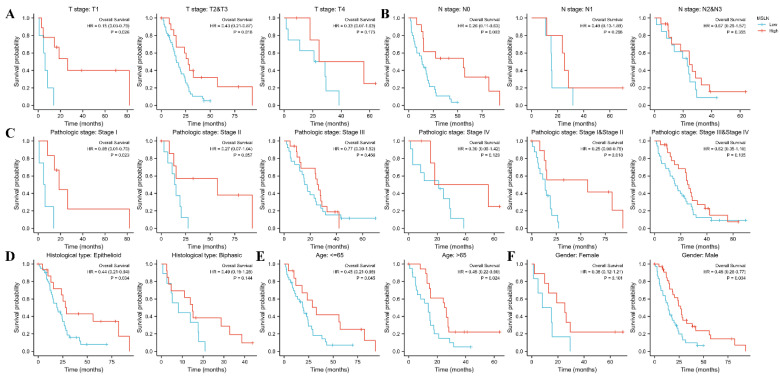
Subgroup prognostic analysis of MSLN gene in mesothelioma. (**A**): Primary tumor stage (T stage); (**B**): MPM regional lymph node metastasis stage (N stage); (**C**): Pathological stage; (**D**): Histological subtypes; (**E**): Age group; (**F**): Gender group.

**Figure 7:**
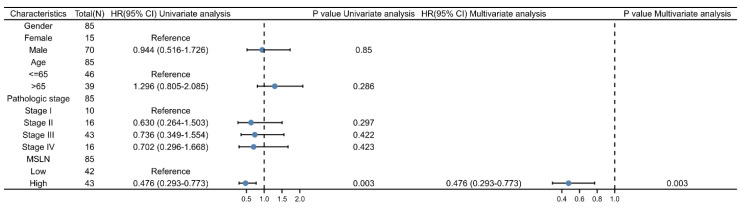
Cox regression analysis between MSLN gene expression and survival time.

Subgroup analysis (Figure 6A) showed that in the T1, T2, T3 subgroups, the higher the MSLN gene expression related to the longer the survival time (Figure 6A), but not in the T4 subgroup. Cox regression results showed that MSLN could be an independent prognostic indicator (HR < 1 and *p* < 0.05) (Figure 7).

In the MPM regional lymph node metastasis stage (N stage, Figure 6B), N0 subgroup showed that the higher the MSLN gene expression level, the longer the patient survival time, and the significant difference in the survival time distribution of the subgroups. N1 and N3 were excluded because of the small number of samples in the database. The Log-rank test results and Cox regression results of the N2 + N3 groupings indicated that there is no differences in the survival time distribution of the groups.

The MPM pathological stage (Figure 6C) was divided into two groups, and the difference in stage I + stage II results was statistically significant, suggesting that the higher the expression level of MSLN in this pathological stage, the longer the survival time of patients. There was no significant difference in the results of stage III + stage IV group.

Histological subtypes of MPM (Figure 6D) are divided into epithelioid, sarcomatoid and biphasic, of which epithelial is the most common. According to histological subtypes, and the results regarding epithelial type, the higher the MSLN gene expression, the longer the patient survival time, and the difference in the results was statistically significant.

According to age, we divided the MPM patients into two groups (Figure 6E). In the groups of MPM patients younger than or equal to 65 years old and older than 65 years old, the higher the expression level of MSLN gene, the longer the survival time of patients, and the difference was statistically significant.

In the gender group (Figure 6F), there was no significant difference in the results of the female group. In the male group, the Log-rank test results showed that the difference in the distribution of survival time was statistically significant, *p* = 0.003. Cox regression results showed that the difference of survival time distribution was also statistically significant, *p* = 0.004. The higher the MSLN gene expression, the longer the survival time of patients.

Fibulin-3 was encoded by EFEMP1 gene. To explore whether this gene is also the same as MSLN gene and showed a positive correlation between the expression level and the survival time of patients with MPM at the molecular level, we analyzed the EFEMP1 gene. Although the results were unsatisfactory, all subgroup analysis showed that the difference was not statistically significant, but Fibulin-3 showed a high accuracy in early diagnosis, suggesting that it can be used as a member of the biomarker combination diagnosis of MPM.

### 3.5. Publication Bias

Asymmetric Deek’s funnel figure test evaluation study of potential publication bias added (Supplementary Figure S3), *p* value is 0.83. This suggests that in this meta-analysis that included research articles, there is no publication bias.

## 4. Discussion

In the present study, a systematic review and meta-analysis based on all the research data of the three biomarkers for the diagnosis of MPM were conducted. Data from 31 studies involving 8750 participants were evaluated. The result showed that Fibulin-3 had the highest diagnostic accuracy in the diagnosis of MPM. In general, the results of this study will help to promote the application and improvement of clinical noninvasive MPM detection methods.

As an aggressive, treatment-resistant tumor, MPM is increasing in frequency throughout the world. For clinicians, MPM is easily missed due to its low incidence and non-specificity. At present, the diagnosis of MPM depends entirely on histopathological examination, the most recommended diagnostic method for MPM requires invasive examination. Since early diagnosis and subsequent intervention are considered to improve the efficacy of the disease, reliable and non-invasive diagnostic tools are urgently needed to shorten the diagnosis delay.

Previous studies focused on many biomarkers such as SMRP, OPN, Fibulin-3, and MSLN, but their diagnostic accuracy of MPM is not optimistic. For SMRP, Gao et al. found that SMRPs detected in pleural effusion (PE) had an unfavorable diagnostic performance with poor SEN (0.69), high SPE (0.90), and AUC (0.76) indicating that the overall accuracy was not as high as expected [21]. Our results are consistent, with pooled sensitivity (0.68) and specificity (0.86), suggesting that SMRP is not a marker for the early diagnosis of MPM.

Considering the heterogeneity of mesothelioma, a single biomarker cannot provide necessary sensitivity and specificity for the clinical practice is indeed possible. Compared with the other two markers, it may be more appropriate to select Fibulin-3 as one of the indicators for combined diagnosis. The data of other markers were analyzed with SMRP as an example, and the value of these markers in the diagnosis of MPM cannot be ignored. Despite the poor sensitivity of SMRPs, regarding sarcomatoid or other types of mesotheliomas, the high specificity of SMRPs can indicate mesothelioma, which provides strong evidence for further invasive examination. At the same time, the diagnostic value of Fibulin-3 is better than SMRP and MSLN, demanding further head-on comparison research.

It is also worth mentioning that today, in the era of precision medicine, it is important to provide clear evidence for targeted therapy or immunotherapy [58,59]. We also paid attention to other biomarkers for the diagnosis of MPM, such as DNA and miRNA, and put forward the idea that they may be combined with proteins to form a specific diagnostic panel for markers to further improve the accuracy of diagnosis. The ideal marker, or a combination of several markers, should be readily available and accurate to avoid false positive results [60]. In healthy subjects, with enough sensitivity to identify MPM subjects, they are able to distinguish between MPM and other diseases. In fact, if a good marker is used for clinical environmental diagnosis (i.e., not for screening) for diagnostic and differential purposes, it should have excellent discrimination ability between healthy people and patients and imaging between different pathology in addition.

The overall prognosis analysis of MSLN gene in mesothelioma showed that the higher the MSLN expression level, the longer the patient’s survival time and the better the prognosis. In the following subgroups, the analysis results showed that the higher the MSLN gene expression, the longer the patient survival: the T2+T3 subgroup in T stage of MPM), the N0 subgroup of MPM N stage, the MPM pathological stage I + stage II group, the male group, the MPM patients aged less than or equal to 65 years old and the subgroup of more than 65 years old. Our findings differ from others’ conclusions that elevated levels of SMRP and MSLN protein assays are associated with worse prognosis [61]. The authors analyzed the possible influencing factors as follows: In MPM, the relationship between the elevated expression of SMRP protein and MSLN protein in patient samples and the expression of MSLN gene is still unclear, and there are still many relationships between protein translation and gene regulation. There is no research-proven mechanism. However, it is certain that the post-transcriptional regulation mechanism of MSLN gene plays an important role. The previous research results only extracted the corresponding proteins from patient samples for detection, or only proposed the phenomenon that the methylation level of MSLN gene was reduced or absent in MPM [62] and did not dig deep into the regulatory mechanism at the molecular level, but they also point out that the direct relationship between MSLN gene methylation and gene expression is currently unclear. Both their study and our analysis were limited by the small sample size, which affected the results of both parties to varying degrees.

Our meta-analysis results suggest that although SMRP and MSLN have inferior diagnostic sensitivity and specificity compared with Fibulin-3, the role of MSLN gene in the prognosis of MPM cannot be ignored. This provides a breakthrough for the diagnosis and treatment of MPM: finding ways to enhance the direct expression of MSLN genes in MPM can significantly prolong the survival of patients. At the same time, we need to continue to explore the relationship between the direct expression of MSLN gene, methylation and the corresponding protein expression, and what kind of changes have occurred in the middle, resulting in the display of completely different prognostic trends at the molecular and protein levels, these findings will contribute to the diagnosis and treatment of MPM.

However, there are still limitations in the process of our study, which are worthy of our consideration and improvement in subsequent studies. The quality of formulations was good in all the studies, but there were significant differences in the number of participants, clinical typing, and diagnostic thresholds. Similarly, blinding, cross-sectional study design, continuous random design, and prospective design also affect the accuracy of diagnosis. Most studies have questions about whether samples remain stable when sent to the laboratory for testing. There are many influencing factors, and they have not been fully studied.

Therefore, it is undeniable that simple biomarker tests still cannot replace invasive examinations such as biopsy. However, alternative biomarker testing based on a combination of several biomarkers may increase related auxiliary information and increase the possibility of making the correct diagnosis. Instead of relying on a single biomarker level, clinicians can use continuous biomarker levels to monitor symptomatic patients.

## 5. Conclusions

Based on the data obtained in this study, we concluded that Fibulin-3 can be used as one of the members of the biomarker combination diagnostic series compared with the other biomarkers. At the same time, the results showed that the higher the MSLN gene expression, the longer the survival time and the better the prognosis of MPM patients.

## Figures and Tables

**Table 1:** Summary of included studies.

First Author	Year	MPM	Non-MPM	Biomarker	Reference Test	Characteristic	TP	FP	FN	TN
Bruce W. S. Robinson [28]	2003	42	228	MSLN	Histology	UP	37	10	7	218
Arnaud Scherpereel [29]	2006	60	23	SMRP	Histology	UP	48	4	12	19
Heather L. Beyer [24]	2007	88	998	SMRP	Histology	UP	46	66	42	932
Alfonso Cristaudo [30]	2007	107	607	SMRP	Histology	UP	73	149	34	458
Francesca Di Serio [31]	2007	24	92	SMRP	Histology	UP	16	7	8	85
Harvey I. Pass [32]	2008	90	236	SMRP	Histology	UP	73	30	17	206
		45	50	SMRP	Histology	UP	34	9	11	41
Monica Amati [33]	2008	22	148	SMRP	Histology	UP	16	15	6	133
Michel M. van den Heuvel [34]	2008	73	156	SMRP	Histology	UP	44	22	29	134
Joost P.J.J. Hegmans [35]	2009	41	48	SMRP	Cytology/histology	UP	34	12	7	36
Jose’ A. Rodríguez Portal [36]	2009	36	326	SMRP	Surgical biopsy, IGB, IHC, fluid cytology	UP	26	91	10	235
Helen E. Davies [37]	2009	24	142	MSLN	Histology	UP	17	14	7	128
Nobukazu Fujimoto [38]	2010	23	73	SMRP	Histology	UP	16	23	7	50
Christophe Blanquart [39]	2012	61	40	SMRP	Fluid cytology and IHC	UP	39	0	22	40
Harvey I. Pass [17]	2012	92	290	Fibulin-3	NR	UP	89	13	3	277
Pier Aldo Canessa [40]	2013	34	70	SMRP	Medical thoracoscopy	UP	20	2	14	68
Paola Ferro [41]	2013	43	59	SMRP	Examination of hematoxilin and eosin stained biopsy sections	UP	30	7	13	52
		43	59	SMRP	Combined with IHC	UP	20	9	23	50
Pier Aldo Canessa [42]	2013	82	120	SMRP	Histology	UP	48	5	34	115
Rosa Filiberti [43]	2013	57	120	SMRP	cytology	UP	42	17	15	103
Clare E. Hooper [44]	2013	25	171	MSLN	Histology	UP	18	21	7	147
Maria Cristiana Franceschini [45]	2014	38	57	SMRP	Basis of clinical signs, imaging data	UP	18	10	20	47
		38	57	SMRP	cytological examination of pleural effusion and histology of pleural biopsies	UP	27	7	11	50
Jenette Creaney [46]	2014	183	1148	MSLN	Cytology or histology	UP	123	57	60	1091
Mohammed A. Agha [47]	2014	25	11	Fibulin-3	Histology	UP	22	2	3	9
Alaa eldin M. Elgazzar [48]	2014	30	30	Fibulin-3	Histology	UP	30	1	0	29
Petr Jakubec [49]	2015	3	236	MSLN	Pathology and morphology	DOWN	2	22	1	214
Michaela B. Kirschner [50]	2015	84	56	Fibulin-3	Histology	DOWN	11	4	73	52
Halide Kaya [51]	2015	43	40	Fibulin-3	Histology	DOWN	42	5	1	35
Melike Demir [52]	2016	42	99	Fibulin-3	Histopathology	UP	37	37	5	62
		42	48	SMRP	Histology	UP	37	16	5	32
Guntulu Ak [53]	2017	95	103	MSLN	IHC	UP	49	23	46	80
		95	103	MSLN	IHC	UP	76	43	19	60
Zhaoqiang Jiang [54]	2017	15	94	Fibulin-3	Pathology	UP	13	2	2	92
		15	74	Fibulin-3	Pathology	UP	13	5	2	69
		15	218	Fibulin-3	Pathology	UP	13	18	2	200
		15	29	Fibulin-3	Pathology	UP	13	7	2	22
Takehiro Otoshi [55]	2017	32	208	SMRP	NR	UP	18	28	14	180
Georg Johnen [56]	2018	26	136	MLSN	NR	UP	6	1	20	135

IGB: image-guided biopsy; UP: up-regulated.

## Supplementary Materials

The following supporting information can be downloaded at: https://www.mdpi.com/article/10.3390/diagnostics12092210/s1, Figure S1: The quality of included studies; Figure S2: The detailed information about the risk of bias and applicability concerns for each included study Quality plot graphically representing the risk of bias (RoB) analysis; Figure S3: Deek’s funnel plot for the studies included in the meta-analysis.
