# Supplementary material for: Systematic Review, Meta-Analysis and Bioinformatic Analysis of Biomarkers for Prognosis of Malignant Pleural Mesothelioma
Source: Diagnostics (Basel). 2022 Sep 12;12(9):2210. doi: 10.3390/diagnostics12092210 (PMC9497920; doi:10.3390/diagnostics12092210)
Supplement: Supplementary file 1 [file diagnostics-12-02210-s001.zip › diagnostics-1886769-supplementary.pdf]

diagnostics

Supplementary

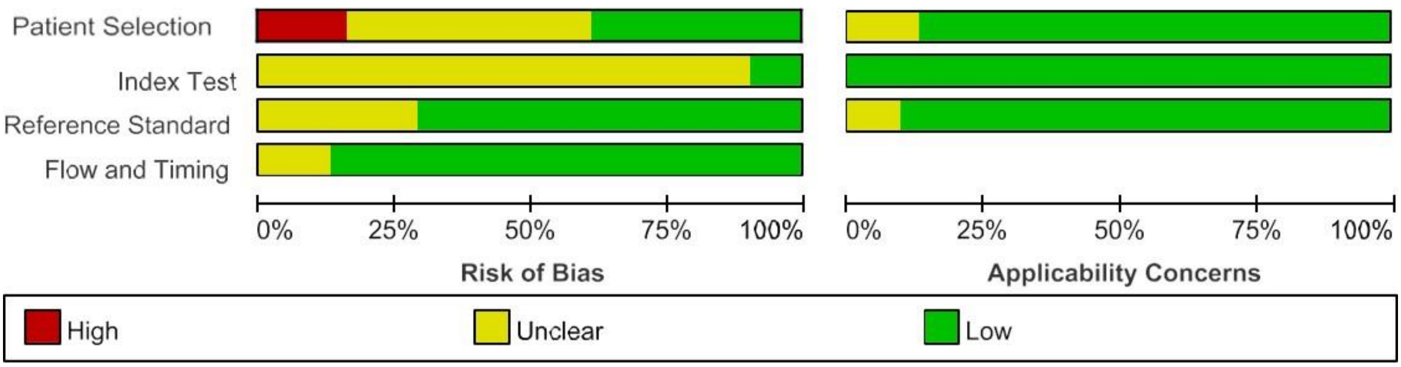

Figure S1. The quality of included studies.

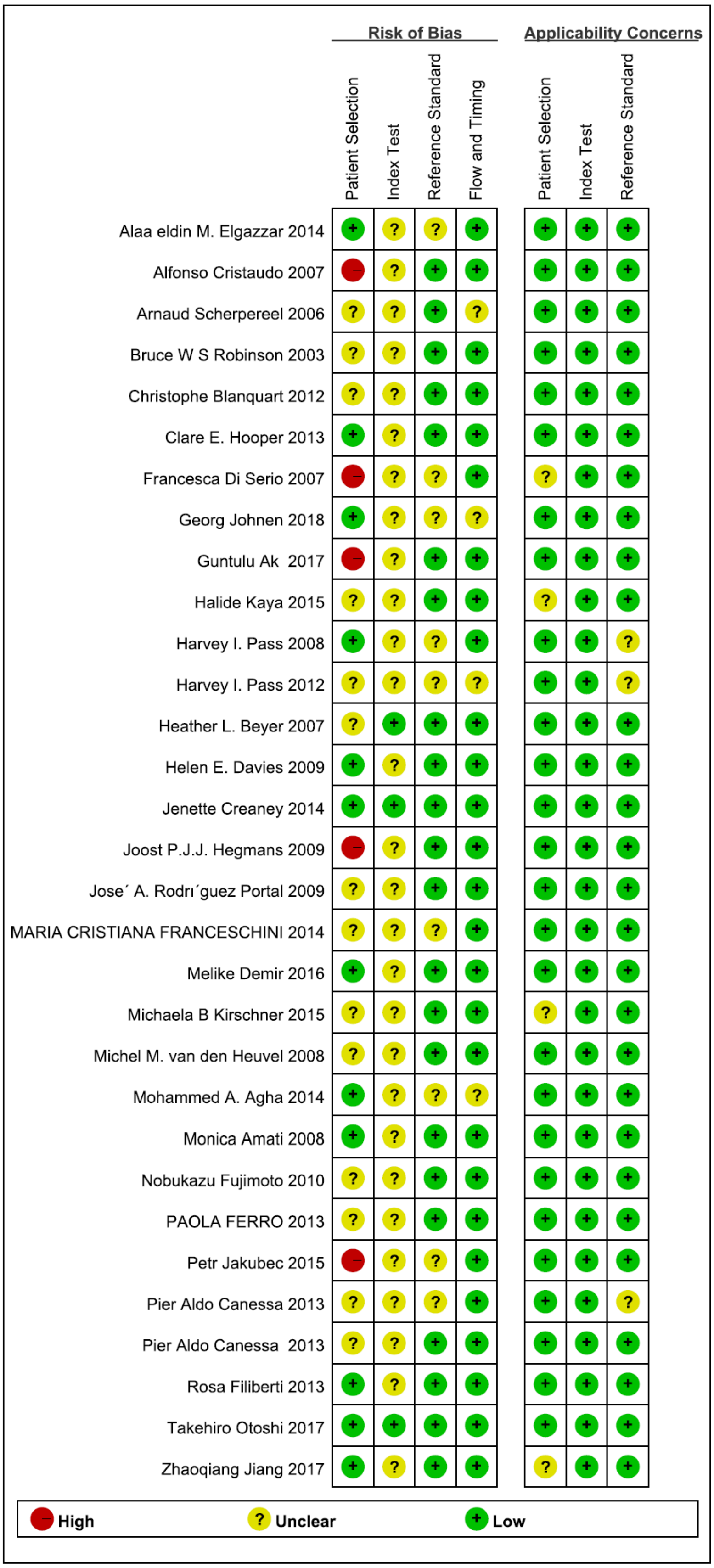

Figure S2. The detailed information about the risk of bias and applicability concerns for each included study Quality plot graphically representing the risk of bias (RoB) analysis.

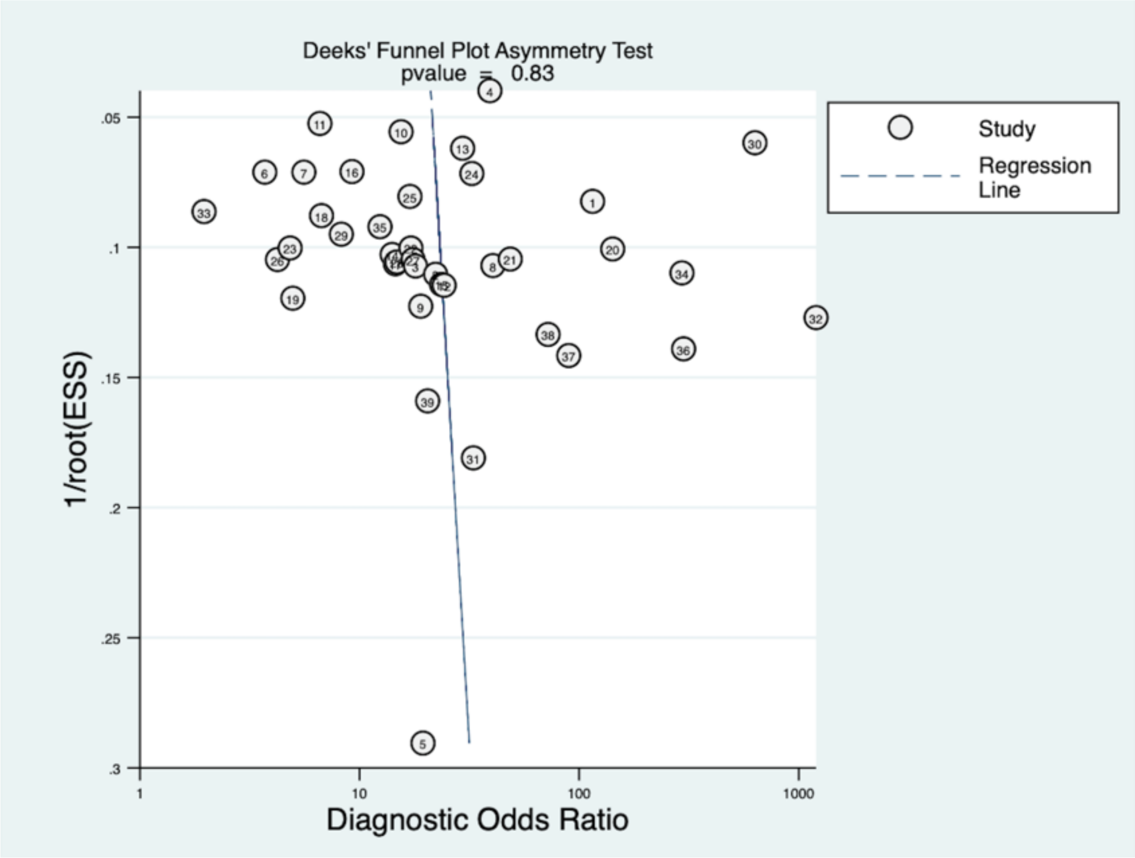

Figure S3. Deek's funnel plot for the studies included in the meta-analysis.
